# Collagen-bound fibrin sealant (TachoSil®) for dural closure in cranial surgery: single-centre comparative cohort study and systematic review of the literature

**DOI:** 10.1007/s10143-022-01886-1

**Published:** 2022-11-02

**Authors:** Alessandro Carretta, Mirka Epskamp, Linus Ledermann, Victor E. Staartjes, Marian C. Neidert, Luca Regli, Martin N. Stienen

**Affiliations:** 1 Department of Neurosurgery, University Hospital Zurich, Zurich, Switzerland; 2 Clinical Neuroscience Center, University of Zurich, Zurich, Switzerland; 3 Machine Intelligence in Clinical Neuroscience (MICN) Laboratory, Zurich, Switzerland; 4 Department of Biomedical and Neuromotor Sciences (DIBINEM), University of Bologna, Bologna, Italy; 5 Department of Neurosurgery, Cantonal Hospital St.Gallen, St.Gallen Medical School, Rorschacher Str. 95, CH-9007 St.Gallen, Switzerland

**Keywords:** TachoSil, Dural closure, Cerebrospinal fluid leak, Infection, Complication, Outcome

## Abstract

Cerebrospinal fluid (CSF) leakage is a well-known complication of craniotomies and there are several dural closure techniques. One commonly used commercial product as adjunct for dural closure is the collagen-bound fibrin sealant TachoSil®. We analysed whether the addition of TachoSil has beneficial effects on postoperative complications and outcomes. Our prospective, institutional database was retrospectively queried, and 662 patients undergoing craniotomy were included. Three hundred fifty-two were treated with dural suture alone, and in 310, TachoSil was added after primary suture. Our primary endpoint was the rate of postoperative complications associated with CSF leakage. Secondary endpoints included functional, disability and neurological outcome. Systematic review according to PRISMA guidelines was performed to identify studies comparing primary dural closure with and without additional sealants. Postoperative complications associated with CSF leakage occurred in 24 (7.74%) and 28 (7.95%) procedures with or without TachoSil, respectively (*p* = 0.960). Multivariate analysis confirmed no significant differences in complication rate between the two groups (aOR 0.97, 95% CI 0.53–1.80, *p* = 0.930). There were no significant disparities in postoperative functional, disability or neurological scores. The systematic review identified 661 and included 8 studies in the qualitative synthesis. None showed a significant superiority of additional sealants over standard technique regarding complications, rates of revision surgery or outcome. According to our findings, we summarize that routinary use of TachoSil and similar products as adjuncts to primary dural sutures after intracranial surgical procedures is safe but without clear advantage in complication avoidance or outcome. Future studies should investigate whether their use is beneficial in high-risk settings.

**Supplementary Information:**

The online version contains supplementary material available at 10.1007/s10143-022-01886-1.

## Introduction

Cerebrospinal fluid (CSF) leakage is an imminent risk of cranial neurosurgery, whenever the dura mater is opened. Possible complications secondary to CSF leakage include persistent CSF fistula, pseudo-meningocele or secondary infection (meningitis/cerebritis), leading to increased morbidity and mortality, prolongation of hospital stay and higher cost of care [1–3]. According to the literature, the incidence of CSF leakage varies between 1 and 30%, depending on the location of surgery (e.g. higher likelihood is commonly reported after posterior fossa surgery), pathology- and patient-related factors and the technique used for dural closure [4–7]. Standard methods of dural closure include use of running or interrupted sutures, which may be supplemented with additional sealants, glues or dural substitutes in order to achieve a water-tight closure [8].

One of the most commonly used commercial products as adjunct in dural closure for neurosurgical interventions is the collagen-bound fibrin sealant called TachoSil® (Takeda Pharmaceutical Company, Tokyo, Japan) [9]. TachoSil is a ready-to-use membrane-like adhesive product, which contains exclusively human coagulation factors [10, 11]. While there is an ongoing development of new sealing materials, there still is no consensus on a standardization of dural closure. Only few clinical studies evaluated outcomes of different closure techniques in a randomized and controlled manner [8, 12]. More comparative data on complication rates and outcomes for different dural closure techniques is needed.

Hence, the aim of this study was to investigate postoperative complication rates and outcomes in patients undergoing craniotomy with or without TachoSil as adjunct to dural sutures. In addition, the results of our own institutional data are complemented by a systematic review of the literature, identifying studies that compared different surgical techniques for dural closure.

## Material and methods

This paper is composed of two parts. In a first step, we conducted a retrospective analysis of a single-centre prospective database, containing demographic, disease- and treatment-specific complication and outcome data of all neurosurgical interventions conducted at the University Hospital of Zurich, Switzerland [13]. In a second step, we conducted a systematic review of the literature identifying and analysing any studies comparing postoperative CSF leakages in patients undergoing craniotomy and appertaining dural closure with or without the use of additional sealing material.

The protocol of this study was designed and carried out according to International Conference of Harmonisation – Good Clinical Practice standards [14]. The data collection was approved by the local Ethics Committee (KEK-ZH 2012–0244) and included patients that signed an informed consent sheet to allow research with their de-identified personal data. The data collection was registered at http://www.clinicaltrials.gov (identifier: NCT01628406) and follows the STROBE recommendation for observational studies.

### Part I — Institutional data collection and analysis

#### Data sources

Our institutional database [15] includes prospectively collected information on patient age and sex, operation date, indication for and type of surgery, lesion location, re-operation, surgeons, operation time and occurrence of a complication [16–18]. Furthermore, standardized scores used to estimate general well-being and function in daily life (Karnofsky Performance Score (KPS)), disability (modified Rankin Scale (mRS)) and neurological status (National Institutes of Health Stroke Scale (NIHSS)) at time points of hospital admission, discharge and 3 to 6 months after surgery are documented in the database [19–21]. The information whether TachoSil was used for closure during any neurosurgical procedure was extracted manually from the billing form for all patients. The extracted data were entered into an ad hoc database for analysis.

#### Inclusion/exclusion criteria

All patients undergoing craniotomy in a timespan of 22 months between January 2018 and October 2019 with opening and closing of the dura mater during the same procedure were included. Interventions involving extensive dural opening without closure, e.g. as for decompressive craniectomies, were not considered. Also, paediatric neurosurgical interventions (e.g. posterior fossa decompression for Chiari malformations) were excluded for their distinct risk profile. Whereas re-operations due to postoperative complications (e.g. wound healing issues, infections, rebleeding) were excluded as index procedures, previous surgery at the same site for, e.g., tumour relapse in neurooncological or aneurysm regrowth in vascular patients, were not considered exclusion criteria as previous literature indicates a similar risk profile [18].

#### Surgical procedure and TachoSil application

All surgeries involved a dural incision and subsequent dural closure during the same procedure. In each case, the dura was closed primarily with sutures (Prolene®, PDS® or Vicryl®, Ethicon Inc., Somerville, NJ, USA). The additional administration of TachoSil was optional and decided by the responsible surgeon according to personal preference. TachoSil is a collagen sponge coated with the human coagulation factors fibrinogen and thrombin. It is a ready-to-use product with simple application onto the surgical site, as it firmly glues to the tissue surface upon contact with blood, other body fluids or saline [11]. The sponge is absorbed by the body within several weeks. In our hospital, TachoSil is applied to the external side of the dura mater, on the suture line, slightly exceeding it with a small overlap with the surrounding pachymeninx on all sides. To achieve optimal and uniform contact, the applied TachoSil is gently pressed onto the dura with moistened surgical gauze.

#### Outcomes and statistical analysis

Relevant demographic data as well as baseline and surgery characteristics were extracted from the database and summarized in a synoptic table. Data are presented as frequencies and percentages for categorical variables and as mean ± standard deviation (SD) or median ± interquartile range (IQR) for continuous variables. Statistical significance was analysed using students’ *t* test for quantitative, the Wilcoxon rank sum test for ordinal and the Pearson chi-squared test for categorical variables, respectively.

As primary end point, the frequency of postoperative complications associated with CSF leakage was compared between the study group (dural closure with TachoSil) and the control group (dural closure without TachoSil). Postoperative complications until 6 months after the index procedure were considered and classified as CSF leakage, CSF fistula, any kind of CSF infection (meningitis and/or cerebritis), wound infection or wound dehiscence. Results are shown as frequencies and percentages for the individual complications, as well as for total complications. For complications, statistical significance was assessed using a chi-squared test. Furthermore, a multivariable logistic regression model was created to adjust for baseline group differences. To include potential confounders in our regression model, variable selection was performed according to the “purposeful variable selection” algorithm described by Bursac et al. [22] Results were analysed for changes after adjustment, and results are expressed as (adjusted) odds ratios (aORs) with 95% confidence interval.

The following secondary end points were considered: KPS, mRS and NIHSS at the time point of hospital admission, discharge and 3 to 6 months postoperative. Statistical significance was calculated using Wilcoxon rank-sum test, and results are presented as *p*-value.

Statistical analyses were performed using R (version 3.6.2, R Foundation for Statistical Computing, Vienna, Austria). The *p*-value was assumed to be statistically significant when ≤ 0.05.

### Part II — Systematic review of the literature

A systematic review of the literature was carried out to identify studies comparing postoperative CSF leakage in patients who underwent cranial neurosurgery involving dural opening and closure with or without the use of a sealing additive. The individual steps of title and abstract screening, full-text review and data extraction were performed independently by two reviewers (ME and LL), and disagreements at any stage were resolved by discussion and consensus. In case of persisting discordance, resolution was achieved through discussion with a third, neutral reviewer. For this part of the study, the Preferred Reporting Items for Systematic Reviews and Meta-Analyses (PRISMA) protocol was applied [23].

#### Search strategy

For the identification of eligible articles, the PubMed/MEDLINE (OVID), Embase (OVID) and Cochrane Library and Scopus (Elsevier) database were searched. The search strategy included combinations of the terms “cerebrospinal fluid leakage, dural defects, postoperative CSF leakage, dural sealant, dural closure, TachoSil, surgical revision, craniotomy, cranial and neurosurgery”, while excluding the terms “transsphenoidal, pituitary and spinal” as those procedures are not of interest for the current research question (Supplemental Table 1). To optimize data mining, word variations and exploded medical subject headings were included whenever feasible. Additionally, reference lists of identified articles were hand-searched to include further studies of interest. The last comprehensive search was conducted on 11 April 2020.

#### Study selection

Only in vivo studies including human subjects and written in English, Italian, Spanish, French, Dutch or German were considered. For a study to be included, patients had to undergo craniotomy with opening and primary closure of the dura mater. Included studies had to compare at least two dural closure techniques, one of which had to include the addition of a sealing additive. Finally, they had to report at least either (a) the proportion of patients with postoperative CSF leakage or (b) other relevant postoperative complications secondary to CSF leakage or (c) the proportion of patients requiring revision surgery because of inadequate dural closure. Detailed in- and exclusion criteria are listed in Supplemental Table 2.

#### Data extraction and assessment of evidence level

The following information was extracted from included publications, whenever available: authors, year of publication, study design, study arms, population, number of patients, outcome measures, results with focus on the primary endpoint and author conclusion. The evidence level of the included studies was determined based on data from the Oxford Centre for Evidence-based Medicine (Supplemental Table 3) [24].

## Results

### Part I — Retrospective single-centre comparative cohort study

A total of 1915 patients were identified, of which 1253 patients undergoing either craniectomy, burr-hole trepanation or endoscopic cranial procedures were excluded or were lost to follow-up within 6 months after surgery and hence omitted from analysis. A total of 662 patients were included: for 352 of them, dural suture alone was performed (control group) and in 310, TachoSil was added in addition to the primary suture (study group). Patient- and disease-specific information, as well as surgery characteristics of the included patients, is summarized in Table 1.

**Table 1:** Baseline table with patient demographics. *KPS* Karnofsky Performance Status, *mRS* modified Rankin Scale, *NIHSS* NIH Stroke Scale

	w/o TachoSil (*n* = 352)	w/ TachoSil (*n* = 310)	*p*-value
Age in years; mean (SD)	55.3 (16.4)	53.9 (15.6)	0.240
Sex, *n* (%)			0.499
Male	143 (40.6)	150 (48.4)	
Female	208 (59.1)	159 (51.3)	
Unknown	1 (0.3)	1 (0.3)	
Type of surgery, *n* (%)			< 0.01
Neuro-oncological	224 (63.6)	255 (82.3)	
Neurovascular	93 (26.4)	19 (6.1)	
Other	35 (9.9)	36 (11.6)	
Lesion location, *n* (%)			0.085
Supratentorial	282 (80.1)	235 (75.8)	
Infratentorial	48 (13.6)	59 (19.0)	
Other	22 (6.2)	16 (5.2)	
Tumor entity, *n* (%)			0.017
Meningioma	61 (17.3)	54 (17.4)	
Low-grade glioma	22 (6.2)	14 (4.5)	
High-grade glioma	30 (8.5)	54 (17.4)	
Metastasis	32 (9.1)	39 (12.6)	
Schwannoma	8 (2.3)	11 (3.5)	
Others	71 (20.2)	83 (26.8)	
Not applicable^a^	128 (36.4)	55 (17.7)	
Reoperation, *n* (%)			0.056
No	286 (81.2)	230 (74.2)	
Yes	63 (17.9)	73 (23.5)	
Unknown	3 (0.9)	7 (2.3)	
Admission scores; median (IQR)			
KPS	90 (10)	90 (10)	0.600
mRS	1 (1)	1 (1)	0.224
NIHSS	0 (1)	0 (1)	0.216

^a^No neuro-oncological procedure performend

#### Analysis of the primary endpoint

Postoperative complications occurred in 7.74% (*n* = 24) of procedures, in which TachoSil was added and in 7.95% (*n* = 28) of procedures with sutures alone (*p* = 0.960; Table 2). Significant differences in disease- and surgery-specific baseline characteristics were also found (Table 1). TachoSil application was more commonly reported in neuro-oncological procedures when compared to neurovascular procedures (*p* < 0.01) and in high-grade glioma and metastasis when compared to other neuro-oncological procedures (*p* = 0.017). Furthermore, TachoSil was also more commonly used in reoperations than in first surgeries, with borderline significance (*p* = 0.056). Therefore, a multivariable logistic regression model was built. In the adjusted model, patients in the study group were 97% as likely as patients in the control group to experience a postoperative complication associated with CSF leakage (aOR 0.97, 95% CI 0.53–1.80, *p* = 0.930; Table 3).

**Table 2:** Incidence of complications following craniotomy and dural closure with (w/) and without (w/o) the use of additional TachoSil

	w/o TachoSil (*n* = 352)		w/ TachoSil (*n* = 310)		Comparison		
	No. of patients	Relative %	No. of patients	relative %	OR	95% CI	*p*-value
CSF leak	1	0.28	2	0.65	0.44	0.04–4.86	0.91
CSF fistula	4	1.13	3	0.97	1.18	0.26–5.33	0.82
CSF infection	2	0.57	2	0.65	0.88	0.12–6.28	0.90
Wound dehiscence	6	1.70	6	1.94	0.88	0.28–2.75	0.82
Wound infection	13	3.69	10	3.23	1.15	0.50–2.66	0.91
Meningitis	2	0.57	1	0.32	1.77	0.16–19.57	0.64
Total complications	28	7.95	24	7.74	0.97	0.55–1.71	0.96

**Table 3:** Multivariable logistic regression analysis of independent predictive effects of the variable of interest (use of TachoSil) and potential confounders on primary outcome (postoperative complications). The model was adjusted for age, sex, type of surgery, location, re-operation, and for clinical admission scores. Tumor entity was not included in the model, as not all procedures were neuro-oncological procedures, which would exclude a large subset of other procedures from analysis

Variable	aOR	95% CI	*p*-value
Use of TachoSil	0.97	0.53–1.80	0.930
Age	1.02	1.00–1.04	0.051
Sex	0.52	0.27–0.96	0.042
Type of surgery (other)	0.96	0.36–3.08	0.946
Type of surgery (neurovascular)	1.14	0.54–2.64	0.745
Lesion location	1.39	0.65–2.81	0.371
Re-operation	1.33	0.64–3.07	0.471
NIHSS	0.96	0.85–1.11	0.551
KPS	1.02	0.97–1.06	0.475
mRS	1.14	0.67–1.98	0.638

#### Analysis of secondary endpoints

In both groups, average scores of function, disability and neurological status showed a slight decline between baseline and 6-month postoperative. There were no significant differences in the change in KPS (− 5.48 vs. − 6.59, *p* = 0.667), mRS (+ 0.37 vs. + 0.01, *p* = 0.182) and NIHSS (+ 0.32 vs. + 0.34, *p* = 0.741) between the study and control group, respectively.

### Part II — Systematic review of the literature

**Fig. 1:**
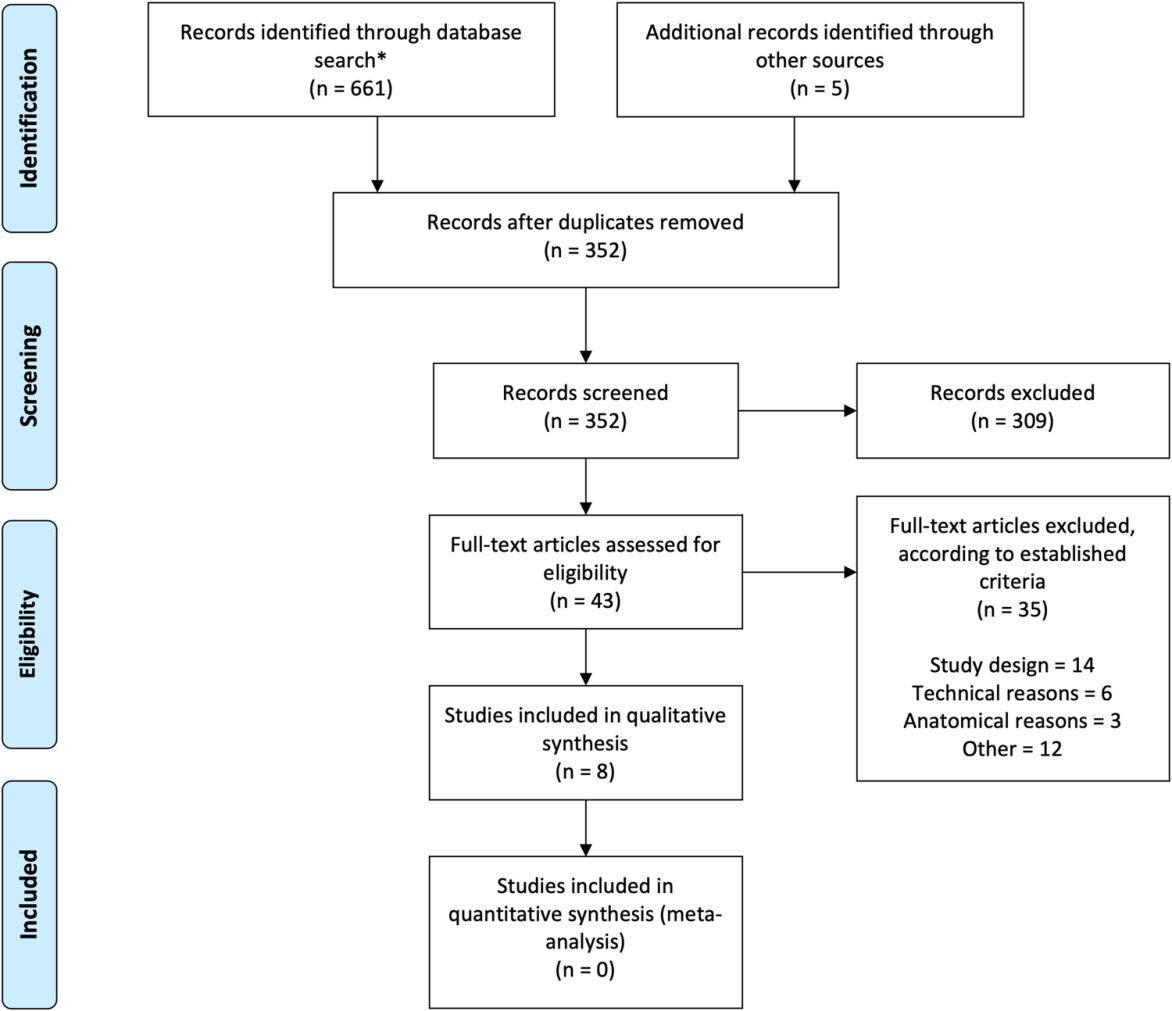
Flow diagram applied to the retrieval and selection of studies included in the systematic review. PubMed/MEDLINE (*n* = 172), Embase (*n* = 243), Cochrane library (*n* = 33), Scopus (*n* = 213)

A flow-diagram for the systematic literature search is provided in Fig. 1. The database search — after removal of duplicates — yielded 352 eligible articles. During title and abstract screening, 309 articles were excluded for not meeting in- or for meeting exclusion criteria. Of the remaining 43 articles, further 35 records were excluded during full-text screening, resulting in eight articles that were used for qualitative synthesis. Excluded papers during full-text screening did not meet the in- or met exclusion criteria, as described in the “Material and methods” section and in Supplemental Table 2. Among those, 14 papers were excluded because of the study design, 6 did not adequately compare the analysed closing techniques, 3 because of anatomical reasons (transsphenoidal or pituitary surgeries) and 12 for other reasons (incomplete studies, duplicates). A comprehensive overview of the included articles is provided in Table 4. Five studies were randomized controlled trials; the remaining were pro- and retrospective cohort studies with control groups. A total of 2045 participants were analysed, of which 1086 were treated with the addition of adjunctive sealant or technique during dural closure (study group), and 959 were treated with standard of care (control groups).

**Table 4:** Summary of included studies with characteristics and results on the primary endpoint

Authors; Year	Study design	Study arms	Population	Nr. of patients	Outcome measure	Primary endpoint	Author conclusions	Evidence level
George et al. 2017 [12]	Multicentre, randomized, prospective, open-label clinical trial	Primary suture plus TachoSil/primary suture alone or in combination (no TachoSil)	Adult patients, undergoing elective non-trauma-related skull base and posterior fossa surgery	Total: 726 Study group: 361 Control: 365	Incidence of CSF leak and pseudomeningocele within 7 weeks after surgery, or treatment failure (change to other surgical technique)	CSF leak occurred less often in the TachoSil group (6.9%) than in the control group (8.2%), the difference was not statistically significant (OR 0.82, 95% CI 0.47–1.43, *p* = 0.485)	TachoSil is at least as effective as current practice and represents a safe and easy to use option for the prevention of CSF leak events after dural closure	1b
Strong et al. 2017 [25]	Multicentre, randomized, prospective, single-blinded clinical trial	Adherus dural sealant (test sealant)/DuraSeal (control sealant)	Adult patients undergoing elective cranial procedures with dural incision	Total: 250 Study group: 124 Control: 126	Proportion of patients free of CSF leak, pseudo-meningocele and unplanned retreatment of the surgical site within 4 months after surgery	Borderline (*p* = 0.056) equality of the test sealant (overall success rate 96.6%) vs. the control group (overall success rate 91.9%) at 45 days follow-up	Adherus dural sealant has equivalent efficacy to other PEG hydrogel sealant products (DuraSeal) for augmentation of primary dural closure in cranial procedures	1b
Green et al. 2014. [26]	Multicentre, randomized, prospective, open-label clinical trial	Suture plus addition of fibrin sealant (EVICEL)/suture alone or in combination (no EVICEL)	Adult patients, undergoing elective craniotomy or craniectomy	Total: 139 Study arm: 89 Control: 50	Intra-operative watertight closure (baseline Valsalva manoeuvre of 20–25 cm H_2_O for 5–10 s), as well as incidence of CSF leakage within 30 days after surgery	Superiority of the study group for intra-operative watertight closure (92.1% vs. 38.0%, OR 24.87, 95% CI 8.53–72.50, *p* < 0.001), similar incidence of postoperative CSF leakage at 30 days (2.2% vs. 2.0%)	Significant better efficacy of dural sealant regarding intra-operative watertight dural closure. No difference in occurrence of post-operative CSF leak	1b
Hutter et al. 2014 [27]	Single centre, randomized, prospective, double-blinded clinical trial	Primary suture plus TachoSil/Primary suture alone	Adult patients, undergoing elective craniotomy with dural incision	Total: 229 Study group: 113 Control: 116	Incidence of CSF leakage, defined as CSF collection or any open CSF fistula within 30 days after surgery	CSF leakage occurred less often in the TachoSil arm (9.7%) than in the control arm (17.2%), albeit the difference was not statistically significant (OR 0.53 95% CI 0.23–1.15, *p* = 0.108)	The use of a dural sealant (TachoSil) leads to a nonsignificant reduction of postoperative CSF leakage within 1 month after surgery	1b
Osbun et al. 2012 [28]	Multicentre, prospective randomized, single-blinded clinical trial	Dural closure augmented with PEG hydrogel/standard of care dural sealing (control)	Patients undergoing elective craniotomy with dural incision	Total: 237 Study group: 120 Control: 117	Incidence of postoperative complications including unplanned reoperations, surgical site infections and CSF leak within 30 days within surgery	No significant (*p* = 0.61) difference for the incidence of postoperative complications between PEG hydrogel (5.8%, CI 2.4–11.6) vs. standard of care (7.7%, 95% CI 3.6–14.1)	PEG hydrogel dural sealant has a similar efficacy to commonly used dural sealing techniques when used as dural closure augmentation in cranial surgery	1b
Arlt et al. 2011 [29]	Single centre, retrospective, case–control study	Sandwich-method (subdural tissue-fleece, epidural TachoSil)/mono-layer (epidural TachoSil)	Patients who underwent microsurgical retrosigmoid resection of vestibular schwannoma	Total: 81, Sandwich: 41 Monolayer: 40	Incidence of postoperative CSF leak (rhinorrhoea or incisional CSF leakage) and postoperative CSF infection within 3 months	No significant (*p* = 0.69) difference in the incidence of postoperative CSF leak between the sandwich technique (7.3%) and the monolayer technique (10%)	The described sandwich technique might show a small advantage compared to the monolayer technique for dural closure after retrosigmoid approach	3b
Than et al. 2008 [30]	Single centre, prospective cohort study, with retrospective control group	PEG dural sealant (test sealant)/fibrin glue (control sealant)	Patients undergoing posterior fossa operation by craniotomy or craniectomy	Total: 200 Study group: 100 Control: 100	Incidence of postoperative complications including incisional CSF leak, pseudomeningocele, wound infection, meningitis and hydrocephalus	No significant difference for total postoperative complications (*p* = 0.4), significantly (*p* = 0.03) less incisional CSF leak in the PEG group (2%) vs. the fibrin glue group (10%)	The use of PEG hydrogel as a dural sealant significantly reduces the occurrence of incisional CSF leak, but not of total postoperative complications	2b
Yoshimoto et al. 1997 [31]	Single centre, prospective, controlled study	Suture plus addition of fibrin sealant (Bolheal, Veriplast P)/suture alone	Patients undergoing craniotomy for unruptured aneurysm	Total: 183 Study group: 138 Control: 45	Incidence of postoperative epidural and subcutaneous fluid collection assessed by CT-scan within 4 weeks after surgery	Extradural fluid collection occurred significantly (*p* < 0.05) less often in the sealant arm (26%) than in the control arm (42%, OR 0.474), CSF collection was often temporary	The use of fibrin glue as an adjunct to dural sutures could be beneficial, but the cost against benefit of its use should be taken into consideration	2b

As a crucial endpoint, the occurrence of postoperative CSF leak after dural closure was compared. All eight studies showed less postoperative CSF leaks in the study group. However, the differences of CSF leak incidence between study and control groups were reported to be statistically insignificant in six of these studies (Table 4) [12, 25–29].

## Discussion

Dural closure remains a critical step in any neurosurgical procedure that involves opening meningeal layers of the central nervous system. Insufficient closure may lead to postoperative CSF leakage, which can result in unanticipated morbidity and prolonged length of hospitalization, thereby resulting in increased healthcare costs [2, 32]. During recent decades, a variety of new sealing materials and surgical techniques have been developed and used in clinical practice. However, robust evidence about their efficacy is scarce due to the paucity of relevant studies. There still is no official consensus on a standardization of dural closure technique in the neurosurgical community [33].

In this retrospective institutional single-centre study, we observed a similar rate of CSF-related complications, with a rate of about 8% in both the experimental and control group (Table 2). To minimize the risk of bias from baseline group differences, multivariable logistic regression models were built without changing the main finding of this study (Table 3). To further investigate the potential clinical relevance of CSF leakage — its potential effect on patient outcome — the pre- to postoperative change of standardized scores of function, disability and neurological status was compared. Again, there was no significant difference in outcome between the study and control group.

It is important to note that for the included cases and procedures, surgeons were allowed to choose for each case whether or not to use TachoSil as an additive to dural suture. In our department, there are surgeons across all experience levels with preferences regarding the use of TachoSil or not, which results in a relatively even distribution of cases with low, moderate or high complexity into the study and control group. Still, we cannot fully exclude a systematic bias resulting from operative experience, as junior neurosurgeons with less developed dexterity and potentially less optimal dural readaptation might be tempted to add TachoSil more frequently than experienced senior neurosurgeons. Hence, the self-contained choice of the dural closure technique by each individual surgeon may be a confounder, where the occurrence of postoperative CSF leakage could result more from surgical experience than from the dural closure technique. It was also for this inevitable limitation of our institutional data that we considered a validation of our findings against the currently available relevant literature mandatory.

The findings of the systematic review (Table 4) mirrored our own institutional data, as none of the included studies found a significant difference in the occurrence of clinically relevant complications resulting from CSF leakage between study and control groups. In a study, significantly less postoperative CSF collection was found in the study group compared to the control group [31]. However, CSF collection was often self-limiting and had no influence on the rate of postoperative complications during follow up. Altogether, the investigated new adjuncts and techniques for dural closure were reported as safe and easy-to-use options for the prevention of CSF leakage after dural closure. In conclusion, none of the studies could show a statistically significant superiority over standard of care dural closure techniques regarding overall postoperative complications due to CSF leakage and rate of patients requiring additional revision surgery. Some authors still concluded that the experimental sealing technique might show advantages for dural closure in certain high-risk settings, e.g. after posterior fossa surgery [29] or in patients with critical comorbidities, such as diabetes [27]. While the use of even more water-tight dural closure techniques may be beneficial under these circumstances, no evidence is currently available to support the standardized use of additional dural sealant in daily clinical routine if the dura is meticulously closed using microsurgical techniques.

We decided to not perform a quantitative synthesis (meta-analysis) from the systematic review, as the available studies showed extreme and unavoidable heterogeneity to in- and exclusion criteria, experimental and control techniques and choice or definition of complications and outcomes. Based on the experience made throughout the systematic review, a critical step towards finding the optimal dural sealing technique in the future may require a better standardization of studies in order to allow comparison of different surgical techniques. We anticipate the use of clinically relevant outcomes only, such as postoperative CSF fistula, wound dehiscence and infection and to exclude often self-limiting and purely imaging-based outcome measures (e.g. subcutaneous CSF collection) without clinical relevance. Furthermore, future studies should ideally record quality outcomes such as length of hospital stay, days spent on the intensive care or intermediate care units, as well as the necessity of revision surgery until 90 days postoperative, besides cost-effectiveness [27].

### Strengths and limitations

This paper contains prospectively collected data with high granularity from a tertiary European neurosurgical department, allowing for robust estimate of the effect size of the experimental technique on complications and clinical outcomes due to the reasonably large sample size. We consider the inclusion of a high number of different surgeons and procedures as a distinct strength, as the findings are more likely to be representative of the real-life clinical scenario and can more easily be transferred to other centres and settings. Moreover, a systematic literature review validates our results against the available literature, building up additional credibility to the reported findings.

Unavoidable limitations are inherent to the retrospective nature of the study. Also, it is likely that there are other parameters that can influence the primary outcome, which, although conceivable, have neither been studied in our own nor in the papers included in the systematic review. Those factors could include significant comorbidities, immune system strength and general capacity for wound healing [27], or elevated preoperative C-reactive protein levels [6, 27]. The limitation regarding the level of experience and selection of additional dural sealant as potential confounder has been discussed above.

### Conclusions and implications of our study

According to own institutional data and in agreement with a systematic review of the current literature, the general use of TachoSil and similar products as adjuncts to primary dural sutures does not seem to reduce CSF-related postoperative complications or improve clinical outcomes. Whether or not additional dural sealant may be beneficial in certain high-risk settings (e.g. posterior fossa surgery, revision surgery) remains unclear and should be the focus of future studies that should ideally include cost-effectiveness analyses.

## Supplementary Information

Supplementary file 1.
