# Supplementary material for: Collagen-bound fibrin sealant (TachoSil®) for dural closure in cranial surgery: single-centre comparative cohort study and systematic review of the literature
Source: Neurosurg Rev. 2022 Nov 2;45(6):3779–88. doi: 10.1007/s10143-022-01886-1 (PMC9663376; doi:10.1007/s10143-022-01886-1)
Supplement: Supplementary file 1 [file 10143_2022_1886_MOESM1_ESM.pdf]

Supplementary Material to **Neurosurgical Review** submission of the following manuscript.

Collagen-bound fibrin sealant (TachoSil®) for dural closure in cranial surgery: single center comparative cohort study and systematic review of the literature.

Alessandro Carretta*[1,2,3,4]; Mirka Epskamp*[1,2,3]; Linus Ledermann[1,2,3]; Victor E. Staartjes[1,2,3]; Marian C. Neidert[5]; Luca Regli[1,2,3]; Martin N. Stienen[5]

[1] Department of Neurosurgery, University Hospital Zurich, Zurich, Switzerland

[2] Clinical Neuroscience Center, University of Zurich, Zurich, Switzerland

[3] Machine Intelligence in Clinical Neuroscience (MICN) Laboratory, Zurich, Switzerland.

[4] Department of Biomedical and Neuromotor Sciences (DIBINEM), University of Bologna, Bologna, Italy.

[5] Department of Neurosurgery, Cantonal Hospital St.Gallen, St.Gallen Medical School, St.Gallen, Switzerland

* The two authors contributed equally to the manuscript and share first authorship.

Correspondence to:

Priv.-Doz. Dr. med. Martin N. Stienen, MD

Fellow of the European Board of Neurological Surgeons (FEBNS)

Department of Neurosurgery

Cantonal Hospital St.Gallen

St.Gallen Medical School

Rorschacher Str. 95

CH-9007 St.Gallen, Switzerland

Tel: +41-(0)71-494-2183

Email: mnstienen@gmail.com

Supplementary Table 1: Detailed search strategy.

**Exact search string:**
("csf leak" OR "cerebrospinal fluid leak" OR "CSF leakage" OR "dural defects" OR "postoperative leakage" OR "CSF collection" OR "CSF fistula") AND ("dural closure" OR "dural opening" OR "tachosil" OR "dural suture" OR "dural sealant" OR "dura sealing" OR "dural repair" OR "fibrin sealant") AND ("neurosurgery" OR "craniotomy" OR "cranial" OR "neurosurgical operations" OR "postoperative complications" OR "surgical revision" OR "durotomy" OR "intradural") NOT ("transsphenoidal," OR "pituitary" OR "spinal")

Supplementary Table 2: Article eligibility and selection criteria.

	**Inclusion criteria**	**Exclusion criteria**
Types of studies	*in-vivo*	*in-vitro*
Types of participants	humans	animals
Types of interventions	craniotomy with dural opening	burr-hole trepanation, trauma-related, endoscopy
Types of comparators	primary suture, addition of sealing additive	absorbable or synthetic tissue grafts
Type of outcome measures	postoperative CSF leak, infection, revision surgery	congenital or idiopathic CSF leak

Supplementary Table 3: Summary of Levels of Evidence of the included studies.

**Level of evidence**	**Study type**	**Included studies**	**Total included patients (study group / control)**
I(b)	randomized clinical trials	5	807 / 774
II(b)	prospective cohort studies	2	238 / 145
III(b)	case control studies	1	41 / 40
